# The COVID-19 pandemic and the challenge of using technology for medical education in low and middle income countries

**DOI:** 10.15694/mep.2020.000074.1

**Published:** 2020-04-23

**Authors:** Dario Cecilio-Fernandes, Maria Cândida Ribeiro Parisi, Thiago M. Santos, John Sandars

**Affiliations:** 1 University of Campinas; 2 Edge Hill University Medical School

**Keywords:** COVID-19, faculty development, online teaching and learning, low and middle income countries, medical education

## Abstract

This article was migrated. The article was marked as recommended.

The current use of technology for medical education in low and middle income countries (LMIC) during the COVID-19 pandemic is not yet reaching its potential. We provide recommendations for LMIC that has focus on a systematic framework that considers both faculty development and developing the skills of students. An enormous challenge for all medical educators, but especially in LMIC, continues to be how to maintain clinical teaching in these extraordinary times.

## Introduction

Over the last 3 months, Covid-19 has relentlessly spread as a pandemic across the world, severely disrupting the lives of the three quarters of the world population that reside within low and middle income countries (LMIC). These economically disadvantaged countries in Latin America, South East Asia and Sub-Saharan Africa have not previously developed healthcare and medical education systems to effectively respond to the current challenges presented by the pandemic.

The major challenge of the COVID-19 pandemic to all countries, including LMIC, has been to their healthcare systems. Prior to the COVID-19 pandemic in LMIC there was already the combination of high population health demand and insufficient resources, with low numbers of skilled health professionals and medical educators. The COVID-19 pandemic has required medical educators across the world to deal with the huge responsibility of rethinking how they can continue to deliver high quality medical education at a time when medical schools are closing face to face teaching due to social isolation strategies and educators have to cope with their enormous clinical responsibilities.

In response to the COVID-19 pandemic, most medical schools across the world, including LMIC, have started to rapidly transfer their curricula from face to face to online delivery using ( McKimm, Gibbs, Bishop and Jones, 2020; Goh and Sandars, 2020). However, we are concerned that there are significant challenges for LMIC that have not been beingfully appreciated by the medical educators in these countries and that the expected outcomes of maintaining high quality medical might not be achieved.

In this Personal View, we will discuss the challenge of using technology for medical education in LMIC, which has increased during the current COVID-19 pandemic, and offer practical advice on how its potential can be fully realised at this time of crisis in healthcare and medical education. Our advice is based on our current experience of a medical school in Brazil, which is typical of many other LMIC, and our previous extensive experience in developing, implementing and evaluating online medical education in LMIC.

## Current experience in Brazil

Following other countries, we have rapidly moved to online teaching and learning, without much online experience in both medical educators and students. The process of moving online has been mainly in two ways. First, educators have continued with the same traditional format as face to face education, maintaining the same schedule and time of the classes, except that this was now online. Often, the classes have continued to be from one to four hours duration. Second, teachers have recorded their lectures, which comprises of uploading PowerPoint presentations and several texts. There has also been little coordination by educators to ensure that there is no overlap in provision.

Educators are spending a lot of time and energy in moving to online education and this has been a top-down process with minimal or no input from students.

Sitting for a few hours in front of a computer is a great challenge for students, especially when having a traditional format class delivered online. Students can easily become distracted since most do not have a dedicated place to study at home or student accommodation and there are also other challenges, such as the need for a laptop and poor internet access or connection, with intermittent episodes of ‘connect and disconnect’ throughout the lecture. More importantly, it is difficult to keep track of which students are actually attending the class.

Faculty development is key to ensure teachers can make effective use of different instructional strategies to engage students, but to date our provision has been mainly focused on basic aspects of online teaching, such as how to create an online discussion room or how to record a lecture, with simple tips on limiting recordings to between 15 and 20 minutes.

A major educational challenge is in relation to the social interaction with other students and educators, but also in clinical contexts, with the patient. In Brazil, like other countries that value social relationships, maintaining verbal and non-verbal communication with online education is essential. Many educators have ignored this aspect when creating their online learning, especially since it is an important aspect of the usual provision of traditional face to face lectures in Brazil.

Training our students in the clinical phase has been an enormous challenge which has yet to be resolved. Students in the clinical phase are in a crucial moment of their training in which they develop their clinical examination and reasoning skills but also communication skills with patients and multi-disciplinary teams, as well as their professional identity formation. In many LMICs, especially in Brazil, communication is a key factor in enhancing the patients’ adherence to treatment, with checking that the patient understands their diagnosis and consequences, and discussion of their social network and finance conditions to deal with their ill health. All these elements are very limited with an online environment. In Brazil, like may LMIC, many doctors on graduation will immediately enter clinical practice with little initial support or graded experience to ensure that they are prepared for the demands of real life clinical practice.

## Responding to the challenge

We fully appreciate the urgency with which many medical schools in LMIC have had to respond to the COVID-19 challenge but it is essential that medical educators can take a more strategic view of using technology for medical education in LMIC. We recommend careful consideration of two main inter-related areas. First, is faculty development of medical educators for effective online delivery of the curriculum and second is the development of student skills for effectively learning in an online environment.

### Faculty development

Effective learning using technology requires careful consideration of the many inter-related factors that include the experience of the educators, the needs and previous experiences of students, the available technology, the learning content, the instructional design to provide activities that enhance learning and the local context, including the culture and available infrastructure resources ( Zaharias and Poylymenakou, 2009). The alignment of these important factors can be practically achieved by using a structured approach, such as the ADDIE framework ( Peterson and Bringing, 2003).

The ADDIE framework has been widely used by educators and highlights the importance of considering the important factors in the Analyse phase to inform the Design and Development phases. An essential aspect of the Implementation phase is Evaluation to iteratively inform any changes that are required in Design and Development. The Analyse phase is key to ensuring that the various factors are considered before there is any commitment to adopting a particular approach for using technology. Several examples will be provided to illustrate how this essential phase has a significant influence on the Design and Develop phases:

• Many LMIC have narrow broadband widths with no reliable internet connectivity or electricity supply and many students only have basic smartphones to access the internet and the online learning content ( Barteit *et al*., 2020; Mittelmeier *et al*., 2019). In these circumstances, the opportunity to access learning content offline, at a time and place that is convenient to the learner, is invaluable, as are opportunities for asynchronous communication.
• The use of podcasts, with or without downloadable videos, have been widely and effectively used in many countries ( Hurst, 2019), including LMIC ( Walsh and De Villiers, 2015), as an effective downloadable educational approach. The provision of downloadable text files to enable reading of content offline may have to be considered in LMIC ( Heller, Strobl and Madhok, 2019). Synchronous discussions by video-conferencing, in which there is real-time discussions between educators and students or between students, can be replaced by asynchronous discussions, such as email or social media ( Heller, Strobl and Madhok, 2019). However, moderation by an educator is required to ensure effective learning ( Salmon, 2020).
• A common response to rapidly provide online content is to simply upload pre-existing PowerPoint presentation or to convert the presentation to a video for online delivery. However, effective learning requires an active process and it is essential to craft the learning experience so that it activates prior knowledge and provides opportunities to check understanding ( Gagne *et al*., 2005). It is also important to avoid a ‘more is better’ approach to providing content so that cognitive overload does not occur, especially when multimedia is being used ( Clark and Mayer, 2016). Even for simple podcasts, the enthusiastic tone of the teacher can determine interest and motivate learning ( König, 2020).

### Student skills development

Developing the skills of students to enhance their learning with the use of technology should not be neglected. Transfer of the curriculum to an online delivery approach can significantly challenge some students in LMIC since they may not have acquired the essential self-regulated learning (SRL) skills through their previous experiences of medical education, which is often didactic. Research suggests that students frequently have under-developed skills in effort regulation, goal setting, self-monitoring of their understanding and progress of learning, time management and help-seeking ( Broadbent and Poon, 2015). These can be developed by crafting the online learning experience using an active process ( Gagne *et al*., 2005).

Students can also begin to actively learn by both creating online content, such as podcasts, but also identifying and sharing online content, such as video guides on practical procedure ( Barteit *et al*. 2020). This active approach can also develop the essential SRL skills required by students ( Broadbent and Poon, 2015).

## Conclusion

In this personal view, we have highlighted that the current use of technology for medical education in LMIC during the COVID-19 pandemic is not yet reaching its potential. Overall, we recommend that this can only be achieved in LMIC by following a systematic framework that considers both faculty development and developing the skills of students. It may be possible to substantially transfer the basic sciences education to high quality online learning but the present, and continuing, enormous challenge to all medical educators is how to maintain clinical teaching, especially in LMIC, where there is an urgent need for qualified doctors.

## Take Home Messages

• The COVID-19 pandemic has caused a major disruption of traditional face to face teaching and learning.

• A huge effort has made to move all face to face activities to online.

• The current use of technology for medical education in low and middle income countries (LMIC) during the COVID-19 pandemic is not yet reaching its potential.

• The lack of faculty development and development of students’ skills may avoid achieving the full potential of online teaching and learning.

• Considering the context and challenges of each country is key to maintain a high quality online teaching and learning.

## Notes On Contributors

**Dario Cecilio-Fernandes** is a researcher in the Department of Medical Psychology and Psychiatry, School of Medical Sciences, University of Campinas and has research interest in assessment and skill acquisition. He is also working with faculty development in the University of Campinas.

**Maria Cândida Ribeiro Parisi** is a medical doctor, researcher, diabetologist and diabetic foot specialist. Cândida has been involved with medical education since the beginning of her career. First, as a preceptor of residents and internals and then as a teacher of a simulation training aimed to teach fourth-year medical students how to care with patients with the diagnostic of diabetes. Currently, she is part of the team that is learning how to use technology for online learning.

**Thiago M. Santos** is an assistant professor of Emergency Medicine at the School of Medical Sciences of the State University of Campinas (UNICAMP), Brazil. He is specialized in Intensive Care Medicine and the Coordinator of the Emergency Intensive Care Unit at UNICAMP’s General Hospital. He is currently developing several projects related to medical simulation, medical education and the use of Point of Care Ultrasonography in critically ill patients.

**John Sandars** is Professor of Medical Education at Edge Hill University Medical School, Ormskirk, UK, and co-chair of the AMEE Technology Enhanced Learning Committee. John was Director of E-Learning in Health in the Evidence for Population Health Unit at the University of Manchester and was a member of the team that developed and implemented the first online UK Master in Public Health programme, with students from across the world. In addition, John has developed and implemented modules on the online Peoples-Uni programme, which has a focus in improving public health in LMIC.
