# Supplementary material for: The COVID-19 pandemic and the challenge of using technology for medical education in low and middle income countries
Source: MedEdPublish (2016). 2020 Apr 23;9:74. [Version 1] doi: 10.15694/mep.2020.000074.1 (PMC10697503; doi:10.15694/mep.2020.000074.1)
Supplement: Peer review history

# Reviewer report: Reviewer response for version 1

**DOI:** 10.21956/mep.20053.r30741

**Published:** 2020-05-02

**Authors:** Subha Ramani

**Affiliations:** 1 Harvard Medical School, Brigham and Women's Hospital

This review has been migrated. The reviewer awarded 4 stars out of 5 I enjoyed reading this article and learning to reflect on challenges to adopting an online learning environment. I compliment the authors for highlighting the challenges of moving to virtual education in a low-resourced environment.This article highlights the fact that many elements, resources and challenges, need to be added to the mix before deciding on a specific technological solution. One size does not fit all.Resources that may be taken for granted in higher resourced settings may not be feasible in lower resourced settings.In this paper, the authors have given very practical solutions to their challenges, which are being faced in many parts of the world today.I agree with their recommendation for student involvement and engagement in the educational process. Students may be much more versatile in use of technology and need to actively contribute to exploring what works and what does not.Faculty development is critical during this transition. Just doing the same lecture with the same slides within a virtual asynchronous or synchronous educational setting is not likely to achieve the learning outcomes. They need training in effectively teaching online, troubleshooting, engaging students and evaluating online programs.A useful contribution to the online learning literature.

Reviewer Expertise:

NA

No decision status is available

# Reviewer report: Reviewer response for version 1

**DOI:** 10.21956/mep.20053.r30740

**Published:** 2020-05-01

**Authors:** Shazia Iqbal

**Affiliations:** 1 Alfarabi College of Medicine Riyadh, KSA

This review has been migrated. The reviewer awarded 4 stars out of 5 Thanks for sharing this interesting article. Authors have discussed the challenge of using technology for medical education in LMIC and offered very practical solutions during the COVID-19 crisis in healthcare and medical education. For example, authors have highlighted the role of Faculty development as a key factor to ensure teachers for making effective use of different instructional strategies to engage students, explained well. Moreover, in the current situation a major challenge maintaining the relation to the social interaction with other students and faculty. Although the challenges for technology-enhanced learning adaptation for faculty and student skills development have been highlighted, there is a requirement to mention multiple factors associated with the educational learning environment essential to adopt online learning/ teaching. The educational learning environment is the sum of personal attributes, social factors, curriculum and physical/virtual space. Although authors have elaborated on the tasks related to personal and social components, there is a need to elaborate on the challenges linked to curricular reforms and developing a healthy virtual learning atmosphere in the current situation. For example, there are multiple challenges related to stress burnout among medical students and faculty members in the current situation that could be addressed briefly with optimal solutions.

Reviewer Expertise:

NA

No decision status is available

# Reviewer report: Reviewer response for version 1

**DOI:** 10.21956/mep.20053.r30742

**Published:** 2020-04-29

**Authors:** Samar Ahmed

**Affiliations:** 1 Ain Shams University Faculty of Medicine

This review has been migrated. The reviewer awarded 4 stars out of 5 Thank you for this excellent reflection in the situation in Brazil. The summative experience offered here is really enlightening and offers a great insight into the transition that almost all countries have been going through. I personally relate to the idea of faculty development to be able to accommodate the existing technology. What is new in this article as well is a reflection On the need for capacity building if students as well. I think this is highly called for and can help in the view into the future of the post covid era planning.

Reviewer Expertise:

NA

No decision status is available

# Reviewer report: Reviewer response for version 1

**DOI:** 10.21956/mep.20053.r30743

**Published:** 2020-04-24

**Authors:** P Ravi Shankar

**Affiliations:** 1 American International Medical University

This review has been migrated. The reviewer awarded 4 stars out of 5 Thank you for an interesting article on teaching-learning in the health sciences during the ongoing pandemic. The pace of spread of the pandemic has been exponential and most schools and students have been caught struggling to cope. As the authors mention in the manuscript most medical schools in the developed world already had functioning learning, student and curriculum management systems and online libraries and were better placed to cope. The authors briefly mention various challenges faculty and students face in switching over to online learning. The issue of narrow bandwidth has been mentioned. Many faculty members may not have the required privacy, resources and bandwidth at home to create and upload sessions. Students as mentioned may be digital natives but may not have been trained to use online educational resources. The authors suggestions on creating online content with less bandwidth requirements are very feasible. Most students may not be having a laptop and may only have a smartphone. So content which can be accessed on a smartphone has to be prioritized. This article adds to the series on teaching-learning during the pandemic.

Reviewer Expertise:

NA

No decision status is available
